# Expression Profiles of Long Non-Coding RNA *GAS5* and MicroRNA-222 in Younger AML Patients

**DOI:** 10.3390/diagnostics12010086

**Published:** 2021-12-30

**Authors:** Djordje Pavlovic, Natasa Tosic, Branka Zukic, Zlatko Pravdic, Nada Suvajdzic Vukovic, Sonja Pavlovic, Vladimir Gasic

**Affiliations:** 1 Laboratory for Molecular Biomedicine, Institute of Molecular Genetics and Genetic Engineering, University of Belgrade, 11042 Belgrade, Serbia; djordje5996@gmail.com (D.P.); natasa.tosic@imgge.bg.ac.rs (N.T.); branka.zukic@imgge.bg.ac.rs (B.Z.); sonja.pavlovic99@gmail.com (S.P.); 2 Clinic of Hematology, Clinical Center of Serbia, 11000 Belgrade, Serbia; zlatko.pravdic@gmail.com (Z.P.); suvajdzic.nada@gmail.com (N.S.V.); 3 School of Medicine, University of Belgrade, 11000 Belgrade, Serbia

**Keywords:** AML, *GAS5*, miR-222

## Abstract

Acute myeloid leukemia (AML) is a heterogeneous malignant disease both on clinical and genetic levels. AML has poor prognosis and, therefore, there is a constant need to find new prognostic markers, as well as markers that can be used as targets for innovative therapeutics. Recently, the search for new biomarkers has turned researchers’ attention towards non-coding RNAs, especially long non-coding RNAs (lncRNAs) and micro RNAs (miRNAs). We investigated the expression level of growth arrest-specific transcript 5 (*GAS5*) lncRNA in 94 younger AML patients, and also the expression level of miR-222 in a cohort of 39 AML patients with normal karyotype (AML-NK), in order to examine their prognostic potential. Our results showed that *GAS5* expression level in AML patients was lower compared to healthy controls. Lower *GAS5* expression on diagnosis was related to an adverse prognosis. In the AML-NK group patients had higher expression of miR-222 compared to healthy controls. A synergistic effect of *GAS5*^low^/miR-222^high^ status on disease prognosis was not established. This is the first study focused on examining the *GAS5* and miR-222 expression pattern in AML patients. Its initial findings indicate the need for further investigation of these two non-coding RNAs, their potential roles in leukemogenesis, and the prognosis of AML patients.

## 1. Introduction

Acute myeloid leukemia (AML) is a hematological malignancy that is characterized by the uncontrolled proliferation and impaired differentiation of early myeloid cells leading to accumulation of immature blast cells in the bone marrow and peripheral blood, thus resulting in hematopoietic failure. AML is the most common acute leukemia in adults, accounting for about 80% of all cases. It is a neoplastic disorder with very poor prognosis, despite the progress made towards discovering the exact etiology of AML. This is due to the fact that the initial treatment protocol is still based on the classification of the patients into risk groups based on the pretreatment karyotype analysis. However, nearly half of the patients do not have any detectable cytogenetic changes. They are referred to as AML with normal karyotype (AML-NK) patients, categorized into the intermediate risk group. Over the years, new molecular markers have been discovered and some of them, like mutations in fms-related tyrosine kinase-3 (*FLT3*), nucleophosmin 1 (*NPM1*), CCAAT/enhancer binding protein alpha (*CEBPA*) gene and runt-related transcription factor 1 (*RUNX1*) gene, have been included into the revised World Health Organization (WHO) classification of myeloid neoplasms and acute leukemia, and European LeukemiaNet (ELN) risk classification system [1,2]. Nonetheless, there is a constant need for discovery of new molecular markers that would lead towards more precise risk stratification and efficient personalized therapeutic protocols.

New high-throughput technologies enabled us to perform transcriptome-wide profiling and to seek new biomarkers of different neoplasms beyond protein-coding genes. Thus, one type of non-coding RNA, long non-coding RNAs (lncRNAs), have been shown to regulate main cell processes like differentiation, proliferation and cell cycle [3]. LncRNAs are defined as transcribed RNA molecules with length over 200 nucleotides. They are not translated into proteins, but are involved in gene regulation, and therefore can be involved in cancer pathology [4].

One of the first lncRNAs that was shown to play a major role in the pathogenesis of cancer is growth arrest-specific transcript 5 (*GAS5*) [5]. The *GAS5* gene, located at the 1q25 locus, is approximately 650 bases in length and is organized in 12 exons and 11 introns. The introns encode 10 box C/D snoRNAs (small nucleolar RNAs) that are involved in methylation processes, and therefore in epigenetic regulation. Exons encode a few splice variants of *GAS5* mRNA, but due to the presence of a stop codon, none of them produce proteins, and the transcripts are degraded through nonsense mediated decay (NMD) pathway [6]. Although the expression level of *GAS5* is increased during growth arrest by serum deprivation, in many types of cancer it was shown that *GAS5* is down-regulated indicating a tumor suppressor function of this lncRNA [7,8].

*GAS5* performs its tumor suppressor function by various mechanisms of action. *GAS5* has the function of a signal molecule and directly participates in the regulation of the p53 signaling pathway. In that way, decreased expression of *GAS5* is associated with cell cycle arrest via increased p53 expression [9]. Also, it can function as a sort of decoy, acting as a molecular “sponge” that binds directly to target RNAs or proteins, thus blocking their downstream functioning [10]. In addition to studying the role of *GAS5* in the pathogenesis of cancer, its potential role as a prognostic marker was also investigated. In numerous types of cancer, it has been shown that reduced *GAS5* expression was associated with unfavorable clinical and pathological characteristics related to advanced stages of the disease, making it a marker for poor prognosis [11,12,13].

Although the role of *GAS5* deregulation has been studied in different types of solid cancers, its impact on the development and prognosis in leukemias, especially in AML, has rarely been investigated. There are only a few studies that are either based on available AML data in The Cancer Genome Atlas database information, or that are dedicated to the existing polymorphisms in the *GAS5* gene and their impact on AML prognosis [14,15]. One study reported that certain variants in the *GAS5* gene can represent risk factors for AML genesis [16]. *GAS5* is of great importance in other types of leukemia, like in certain types of B-ALL characterized by hyperdiploidy or the presence of *TCF3*/*PBX1* rearrangement, but its significance is related to the treatment of ALL, that is, to the administration of glucocorticoids in therapy protocols [17]. In the case of childhood ALL, it has been shown that the *GAS5* expression level can be a predictor of therapy response in the first phase of treatment, remission induction [18].

In addition, one of the main proposed mechanisms with which *GAS5* exerts its tumor suppressor role is through acting as an endogenous sponge for the oncogenic miR-222 [19]. Overexpression of miR-222 has been widely detected across many malignancies, including AML, and has been associated with tumor stage, tumor type and metastasis [20]. The discovery that *GAS5* negatively regulates miR-222 activity was first reported in glioma, and it was later confirmed in gastric cancer and recently in B ALL as well [21,22,23]. Furthermore, it was shown that decreasing miR-222 expression through *GAS5* leads to higher expression of an important miR-222 target, the tumor suppressor PTEN, that plays a significant role in cell growth, apoptosis, and migration [19,22].

This is the first study to analyze *GAS5* expression patterns in a cohort of younger AML patients (<65 years) and examine its potential influence on the prognosis of the disease. In addition, we separately investigated prognostic significance of *GAS5* expression in AML-NK group of patients, taking into account the influence of the miR-222 expression and other already established molecular markers of AML.

## 2. Materials and Methods

### 2.1. Patients and Therapy Protocol

A total of 94 bone marrow samples were collected from younger AML patients (<65 years) diagnosed at the Clinic of Hematology, Clinical Center of Serbia, as well as from 14 healthy controls (bone-marrow donors). A total cohort of AML patients consisted of 75 de novo AML cases, 14 were secondary AML (sAML) patients and 5 were therapy-related AML (tAML) patients. Research was conducted in accordance with the ethical standards of the World Medical Association’s Declaration of Helsinki. The study was approved by the Ethics Committee of the Clinical Center of Serbia, and written informed consent was obtained for all patients. Diagnostics was done based on the cytomorphology, immunophenotype using flow-cytometry and cytogenetic analysis. Cytomorphological analysis implies a marrow or blood blast count of 20% for the diagnosis of AML, except for AML with recurrent cytogenetic abnormalities. Cytogenetic analysis was undertaken by conventional band methodology, while molecular analysis was done using PCR and RT-PCR, and WHO classification was assigned [1]. Mutational analyses of *FLT3* and *NPM1* genes were analyzed as previously described [24]. Prognostic classification of AML patients was done according to European LeukemiaNet (ELN) recommendations [2]. All patients received induction chemotherapy with daunorubicin and cytarabine according to the protocol 3 + 7, followed by three consolidation cycles of high/intermediate doses of cytarabine [2].

### 2.2. Growth Arrest-Specific Transcript 5 (GAS5) and MiR-222 Expression Level Analysis

Mononuclear cells were isolated from bone marrow samples of AML patients and healthy controls using Ficoll-Paque Plus solution (GE Healthcare, Buckinghamshire, UK) according to the manufacturers’ protocol. Mononuclear cells were stored in TRI Reagent (Ambion, Thermo Fisher Scientific, Waltham, MA, USA) and kept at −80 °C until RNA isolation. Total RNA was isolated using manufacturer protocol. Concentration and purity of total RNA was determined on NanoVue™ Plus Spectrophotometer (GE Healthcare, Buckinghamshire, UK).

For the expression analysis of *GAS5*, one microgram of total RNA was used for the cDNA synthesis using RevertAid Reverse Transcriptase (Thermo Fisher Scientific, Waltham, MA, USA). Real-time PCR was performed on 7900HT Fast Real-Time PCR System (Applied Biosystems, Foster City, CA, USA). We have performed PCR using 1µ L of cDNA (20 ng RNA equivalent) with TaqMan^®^ Universal Master Mix II (Applied Biosystems, Foster City, CA, USA), and TaqMan^®^ Gene Expression Assay for *GAS5* (Hs03464472_m1 ) and for GAPDH (Hs99999905_m1) as endogenous control (Thermo Fisher Scientific, Waltham, MA, USA). All samples were run in duplicate. Relative quantification analysis was performed using comparative ddCt method, using healthy controls as calibrator, meaning *ddCt = dCtsample – dCthealthy control* (median). ROC curve analyses using on-line program “Cut-off finder” [25] was applied for the identification of optimal cut-off value for discriminating between *GAS5*^high^ and *GAS5*^low^ expression.

For the expression analysis of miR-222, 60 ng of total RNA was used for the cDNA synthesis using RevertAid Reverse Transcriptase (Thermo Fisher Scientific, Waltham, MA, USA) and specific RT primers from TaqMan^®^ Gene Expression Assays for hsa-miR-222 (002276) and RNU44 (001094) (Thermo Fisher Scientific, Waltham, MA, USA). Real-time PCR was performed on 7900HT Fast Real-Time PCR System (Applied Biosystems, Foster City, CA, USA), using TaqMan^®^ Gene Expression Assays for hsa-miR-222 (002276) and RNU44 (001094), as well as TaqMan^®^ Universal Master Mix II (Applied Biosystems, Foster City, CA, USA). The level of miR-222 expression was calculated relative to RNU44 expression using the comparative ddCt method, and healthy controls as a calibrator.

### 2.3. Statistical Analysis and Definition of Clinical Endpoints

Data is presented as medians with range or as absolute numbers with percentages. Differences in continuous variables were analyzed using the Mann–Whitney U test for distribution between two groups. Analyses of frequencies were performed using the χ^2^ test for 2 × 2 tables, or the Fisher exact test for larger tables. Survival analysis was performed by the Kaplan–Meier method, and differences in survival distributions were evaluated using the Log-Rank test.

Overall survival (OS) was calculated from the first day of therapy to death or last visit. Patients undergoing hematopoietic stem cell transplantation (HSCT) were censored at the time of transplantation (23 patients underwent HSCT). Disease-free survival (DFS) for patients who had achieved complete remission (CR) was measured from the date of CR to relapse/death/last follow-up.

The statistical analyses were performed using the R version 4.1.2. (R Core Team, Vienna, Austria) For all analyses, the *p* values were 2-tailed, and *p* < 0.05 was considered statistically significant.

## 3. Results

### 3.1. GAS5 Expression Level in De Novo Acute Myeloid Leukemia (AML) Patients

The expression level of the *GAS5* gene was first evaluated in a cohort of 94 AML patients: 5 patients were therapy-related AML (tAML), 14 were secondary AML (sAML), and 75 of them were de novo AML patients. Median expression level of the entire cohort of patients was 0.59 (0.02–6.81), which was significantly lower than the level detected in healthy individuals (median 1.40, range 0.77–2.42) (*p* < 0.01).

**Figure 1:**
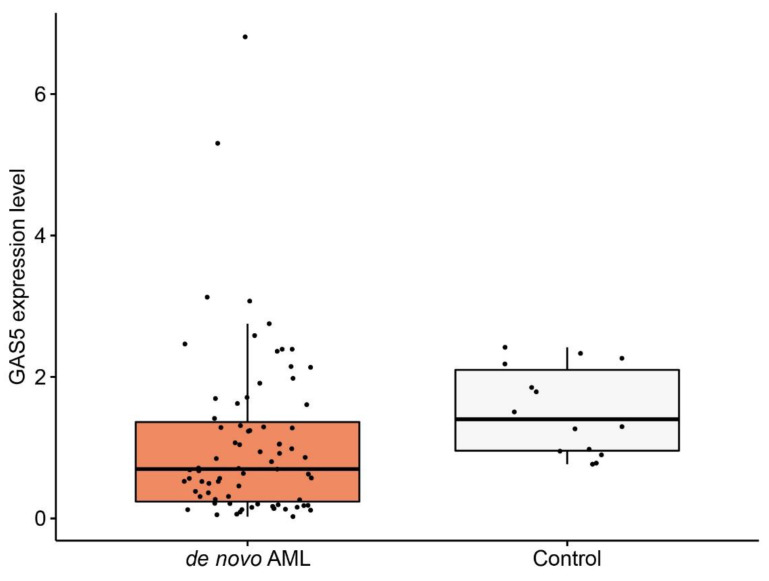
Box-plot representation of growth arrest-specific transcript 5 (*GAS5*) expression detected among de novo acute myeloid leukemia (AML) patients (*n* = 75) and healthy controls (*n* = 14).

Moreover, in comparison to healthy controls, *GAS5* expression level was lower in each of the aforementioned groups of patients individually. In tAML median expression was 0.19 (0.06–1.03) (*p* < 0.01), in sAML it was 0.32 (0.11–3.62) (*p* < 0.01) and in de novo AML it was 0.70 (0.02–6.81) (*p* < 0.01) (Figure 1).

Due to the size of individual groups, further analysis was limited only to de novo AML patients. We used ROC curve analysis to calculate the ‘‘cut-off’’ value for discrimination between AML patients and healthy controls. The *GAS5* expression level of 0.88 was determined as the most predictive one (AUC = 0.73, Sensitivity = 85.7%, Specificity = 57.3%, *p* < 0.01). Using this value, patients were divided into low *GAS5* expression (*GAS5*^low^) and high *GAS5* expression (*GAS5*^high^) group. At diagnosis, 58% of patients (43/75) had low *GAS5* expression. Analyzing the association of *GAS5* expression level with clinical characteristic of de novo AML patients, we found that *GAS5*^low^ patients had significantly higher Lactate Dehydrogenase (LDH) levels in comparison to *GAS5*^high^ patients (*p* = 0.04). Also, *GAS5*^low^ status was significantly associated with adverse prognosis since 15/19 (79%) adverse risk patients had a low *GAS5* expression level (*p* = 0.04) (Table 1).

### 3.2. Prognostic Significance of GAS5 Expression Level in De Novo AML Patients

**Figure 2:**
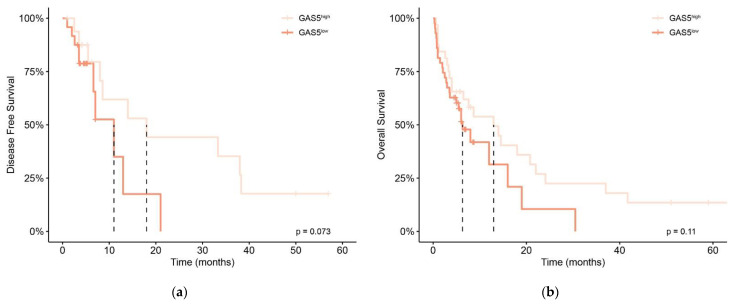
Kaplan–Meier analysis of disease-free survival (DFS) and overall survival (OS) in de novo AML patients according to *GAS5* expression status; (**a**) analyses of DFS between *GAS5*^high^ patients (*n* = 17, 6 censored) and *GAS5*^low^ patients (*n* = 24, 10 censored) (11 vs. 18 months median, Log-Rank = 3.2, *p* = 0.07). (**b**) analyses of OS between *GAS5*^high^ patients (*n* = 32, 8 censored) and *GAS5*^low^ patients (*n* = 43, 13 censored) (6.3 vs. 13 months median, Log-Rank = 2.6, *p* = 0.11).

In our cohort of de novo AML patients, the CR rate was 55%. Among *GAS5*^low^ patients induction treatment failure was more frequent compared to *GAS5*^high^, but without significance (56% vs. 44%, *p* = 1.0). Although the duration of DFS in *GAS5*^low^ patients was significantly lower than in *GAS5*^high^ patients (*p* = 0.02), survival analysis failed to confirm this finding. Nonetheless, a trend towards lower DFS in *GAS5*^low^ patients was evident (11 vs. 18 months median, Log-Rank = 3.2, *p* = 0.07) (Figure 2a). Similarly, the OS duration in *GAS5*^low^ patients was also significantly lower (*p* = 0.03), but survival analysis showed no significant difference in OS between *GAS5*^low^ and *GAS5*^high^ patients (6.3 vs. 13 months median, Log-Rank = 2.6, *p* = 0.11) (Figure 2b).

### 3.3. GAS5 Expression Level in AML-NK Patients

Since AML-NK patients represent the largest subgroup of adult AML patients, and since it is defined as a group with intermediate risk, we wanted to examine whether the analysis of *GAS5* expression can contribute to a more accurate risk stratification. Our cohort consisted of 39 AML-NK patients, and 23 of them (59%) had *GAS5*^low^ status (Table 2). We analyzed the association of *GAS5* expression level with other molecular markers such as *FLT3*-ITD and *NPM1* mutational status (accountable for poor and favorable prognostic influence, respectively) but we could not detect whether these mutations were significantly associated with either *GAS5*^low^ or *GAS5*^high^ patient group (*p* = 0.32, and *p* = 0.21) (Table 2). Survival analysis showed no statistically significant difference in DFS between *GAS5*^low^ and *GAS5*^high^ groups (11 vs. 18 months, Log-Rank = 1.6, *p* = 0.2), and similar results were obtained when we analyzed OS in these two groups (8 vs. 7 months, Log-Rank = 0.2, *p* = 0.6) in AML-NK patients.

### 3.4. Prognostic Significance of MiR-222 Expression Level in AML-NK Patients

To further examine the influence of *GAS5* expression level on prognosis of AML-NK patients, we decided to investigate the expression level of miR-222, a micro-RNA whose oncogenic function is known to be involved in *GAS5* expression and function. The median expression level of miR-222 in AML-NK patients was 5.77 (range 0.08–17.13), which was significantly higher compared to the median expression level found among healthy controls (0.89, range 0.76–1.47; *p* = 0.01). Median expression level was used to divide patients into miR-222 high (miR-222^high^) and miR-222 low (miR-222^low^) expression groups. No significant associations were detected between miR-222 expression status and other prognostic molecular markers. Also, survival analysis showed no statistically significant difference in DFS between miR-222^high^ and miR-222^low^ groups (13 vs. 11 months, Log-Rank = 0.1, *p* = 0.8), nor in OS (11 vs. 14 months, Log-Rank = 0.3, *p* = 0.6) in AML-NK patients.

We detected that 68% of miR-222^high^ patients had *GAS5*^low^ status. In order to investigate the possibility of combined influence of these two ncRNAs on the prognosis and outcome of the disease, in further analysis we defined the *GAS5*^low^/miR-222^high^ group and compared it with other groups that did not meet this criterion. However, survival analysis showed no statistically significant difference in DFS between the *GAS5*^low^/miR-222^high^ group and other patients (13 vs. 11 months, Log-Rank = 0.2, *p* = 0.7). The same goes for the duration of OS within this group of patients that was not significantly different compared to others (16 vs. 6 months, Log-Rank = 0.1, *p* = 0.8).

## 4. Discussion

Although the genetic and epigenetic background and associated cytogenetic and molecular genetic markers have significantly contributed to the understanding of AML biology, as well as to improvement of diagnosis, prognostication, and monitoring of the clinical course of disease, the pathogenesis of AML still remains obscure. Consequently, the majority of AML patients cannot be cured by currently available therapy.

Therefore, many studies are conducted aiming to elucidate the relevant pathogenic mechanisms in AML. The ultimate goal is to discover novel biomarkers and therapeutic targets. In recent years non-coding RNAs, such as, microRNAs and lncRNAs have been postulated as new candidates to be considered as markers relevant for AML, since their role in gene expression regulation has been demonstrated [26,27,28]. Since variants in genes for non-coding RNAs are rare, alterations in their expression levels are a prominent factor governing their effects [29].

The role of lncRNA *GAS5* and miR-222 in pathogenesis of AML has been anticipated only in a few studies [14,30]. Thus, the idea of their clinical use is even more hypothetical. Our study contributes to knowledge concerning AML in this respect. This is the first study enrolling AML patients which intends to evaluate potential clinical application of lncRNA *GAS5* and miR-222 expression.

Our study included a total of 94 clearly stratified younger (<65 years) AML patients, including 75 de novo AML, 14 secondary AML and 5 therapy-related AML patients. We found that, compared to healthy individuals, *GAS5* expression was underexpressed in all these groups of AML patients. This is consistent with previous studies undertaken in different types of solid cancer, as well as in B lymphocytic leukemia. [17,31] This aligns with the hypothesized role of *GAS5* as a tumor-suppressor lncRNA which inhibits proliferation and promotes apoptosis [8,32]. Furthermore, when we analyzed the prognostic impact of *GAS5* expression among de novo AML patients we found that low *GAS5* expression is predominantly detected among patients with an adverse prognosis. This relation of *GAS5*^low^ status with adverse prognosis is consistent with studies investigating *GAS5* in solid tumors, where decrease in *GAS5* expression is associated with characteristics of advanced stage tumor, like tumor size and lymph node and distal metastases [33,34]. This adds to the idea that reduced *GAS5* expression has the same effect in AML as in other types of cancer [31]. Two patients have shown higher levels of *GAS5* expression compared to the other patients. While it is hard to conclude the cause of this, it should be pointed out that these patients have a complex karyotype.

In previously published studies, the prognostic impact of *GAS5* expression in AML was analyzed in publicly available datasets, like the Cancer Genome Atlas, where contrary to our results, *GAS5* overexpression was linked to poor cytogenetic prognosis [14]. The other study uses the GEO GSE12417 dataset and reports a link between *GAS5* overexpression and shorter OS, therefore, it proposes that *GAS5* may have an unexplored proto-oncogenic role. The same authors also suggest that a polymorphism in the *GAS5* promoter leads to higher *GAS5* expression, which in AML is associated with longer platelet recovery, a poor prognosis marker [15]. Contrary to these findings our study indicates that patients with lower *GAS5* expression tend to have shorter durations of DFS, and OS, which would be more in line with the effect of low *GAS5* expression in other cancers [31]. However, when we analyzed AML-NK patients separately *GAS5* expression level did not significantly influence the survival duration. Also, within AML-NK group of patients *GAS5* expression level was not associated with already established prognostic molecular markers, like *NPM1* and *FLT3*-ITD mutational status. Because of the fact that our research is the first in which the impact of *GAS5* expression on duration of DFS and OS in AML was directly investigated, the final conclusion about prognostic significance of *GAS5* expression could be elucidated only through larger studies.

One of the better studied mechanisms of lncRNA *GAS5* action is negative regulation of the oncogenic miR-222 by acting as an endogenous sponge, which was first proven in glioma [21]. The same *GAS5*/miR-222 axis was later shown in gastric cancer, papillary thyroid carcinoma, and most recently in B lymphocytic leukemia [19,22,23]. We analyzed the expression of miR-222 in our AML-NK group of patients and found that it was significantly overexpressed compared to healthy controls. This is in accordance with a previous study that analyzed comprehensive miRNA expression patterns in AML, as well as with studies on miR-222 in other types of cancer [20,35]. However, miR-222 overexpression did not show prognostic significance in our AML-NK group. This is consistent with recent investigation of miR-222 expression, based on data from the Cancer Genome Atlas that also found that high miR-222 expression did not have significant impact on prognosis, and contrary to the oncogenic role of miR-222, the authors reported that high miR-222 expression seemed to indicate a tendency towards longer survival [36]. Because of the reported negative correlation between *GAS5* and miR-222 expression, we performed analysis of the combined influence of *GAS5* and miR-222 expression level on prognosis in AML-NK patients. Nonetheless, survival analysis of *GAS5*^low^/miR-222^high^ vs. other AML-NK patients did not show any statistical significance. These results may be in part due to one of the major targets of miR-222 identified in other malignancies, PTEN, not being as involved in AML development as it is in other malignancies, although further studies on larger cohorts are needed for a conclusive result [19,22,37].

We believe that the results obtained in our study are significant, regardless of the relatively small sample size. Our cohort of AML patients was selected to include only younger patients (<65 years). In that way we tried to avoid the influence of treatment related mortality on the analysis of the prognostic significance of *GAS5* and miR-222. Treatment related mortality is primarily associated with older AML patients resulting in in worse prognosis in this age group. However, overtreatment of younger AML patients is also a common problem [38]. In order to prevent treatment toxicity in younger AML patients, a more comprehensive molecular characterization of each patient individually is needed. Our cohort of patients was tested only for basic molecular markers (cytogenetics, molecular analysis for recurrent translocations and *FLT3*-ITD and *NPM1* mutations). We suspect that our results on *GAS5* and miR-222 expression pattern in younger AML patients would be more clearly defined in their effects on clinical parameters, if the analysis were to be performed in patients who were extensively characterized on a molecular level (i.e., using targeted NGS for most common leukemia-associated mutations), as well as if the impact of other significant variables was grouped in a multivariate approach.

We also believe that our study is important because of the selection of non-coding RNAs that we analyzed. Namely, there is a growing number of studies in which the importance of *GAS5* and miR-222 is related to therapy response, and it has also been shown that *GAS5* and miR-222 themselves can be a target for the design and application of new therapies.

Through identification of the signaling pathways related to *GAS5* and miR-222 a possibility for targeted innovative therapy for AML patients has emerged. Since down-regulation of *GAS5* is a hallmark of different types of cancer, induction of *GAS5* overexpression could aid in regression of the tumor. It was shown that overexpression of *GAS5* leads to decrease of miR-544 expression by overexpression of runt-related transcription factor 3 (*RUNX3*), thus supporting increased activated NK cell-mediated cytotoxicity [39]. Moreover, the expression of *GAS5* is related to mTOR signaling pathway, which makes it a mediator of cytotoxic and cytostatic effects of rapalogues in mantle cell lymphomas [30]. Recently, *GAS5* has emerged as a potential target for cancer immunotherapy. Upregulation of its expression can increase anti-tumor response. It is indicated that *GAS5* has a role in increased NK cell cytotoxicity and that it is also involved in initiation and phagophore nucleation [40]. All of this shows that *GAS5* could potentially be an important therapeutic target in AML as well.

Similarly, miR-222 has become a subject of study for potential therapeutic targeting. It is well documented that miR-222-3p plays various roles in the initiation, progression, metastasis and treatment response of cancer. It is involved in the regulation of multiple tumor signaling pathways, such as PI3K/AKT, PTEN, JAK/STAT, TRPS1/ZEB1 and EMT [36]. Several studies have shown that its expression could be a target for innovative cancer therapeutic approaches [36,41,42,43].

In the era of next-generation medicine, treatment of hematological malignancies, including AML, will be personalized. This can be achieved by utilizing high throughput technologies to characterize patients’ specific multiple “oms” landscape. Personalized treatment protocols will certainly consider non-coding RNAs as molecular markers for individualized drug selection and dosage profiling, as well as for design of novel, molecular targeted drugs. Integrated pharmacomics will open the doors to personalized oncology.

## 5. Conclusions

Recently, the role of non-coding RNAs in cancer pathogenesis has been the focus of researchers. LncRNA *GAS5* and miR-222 are the most studied as potential markers clinically relevant in many malignancies. *GAS5* and miR-222 can also be targets for new therapies. This is the first study focused on examining *GAS5* and miR-222 expression pattern in AML patients. Our results showed that *GAS5* expression level in younger AML patients was lower compared to healthy controls, and it was related to adverse prognosis. In the AML-NK group, patients had a higher expression of miR-222. Complete understanding of AML pathology will be achieved when the puzzle of its “oms” background is solved. There is no doubt that *GAS5* and miR-222 are pieces of that puzzle. Therefore, our initial findings indicate the need for further investigation of these two non-coding RNAs, their potential roles in leukemogenesis, and the prognosis of AML patients.

## Figures and Tables

**Table 1:** Clinical characteristics of de novo AML patients stratified by the level of *GAS5* expression.

Parameter	Total (*n* = 75)	*GAS5* Expression		*p*
		*GAS5*^high^ (*n* = 32)	*GAS5*^low^ (*n* = 43)	
**Sex**				0.78
Male (%)	42 (56)	19 (45)	23 (55)	
Female (%)	33 (44)	13 (39)	20 (61)	
**Age**, years, median (range)	50 (18–62)	51 (24–62)	48 (18–62)	0.44
**WBC (White Blood Cells) count**, ×10^9^/L, median (range)	17.1 (1–348.8)	9.8 (1–183.7)	22.3 (1.2–348.8)	0.09
**Hemoglobin** (g/L), median (range)	97 (2–166)	98.5 (2–153)	97 (24–166)	0.62
**Platelets** (×10^9^/L), median (range)	54 (1–422)	48.5 (12–216)	54 (1–422)	0.59
**LDH** (U/L), median (range)	287.5 (153–4169)	175 (153–4196)	462.5 (2–2904)	0.04
**Peripheral blood blast** (%) median (range)	16 (0–98)	13.5 (0–92)	21 (0–98)	0.43
**Bone marrow blasts** (%) median (range)	63 (21–97)	60.5 (21–97)	67(21–97)	0.81
**CD34** (%)				0.31
present	46 (61)	20 (43)	26 (57)	
absent	22 (39)	6 (27)	16 (73)	
**FAB** (%)				0.15
M0	6 (8)	3 (50)	3 (50)	
M1	10 (13)	5 (50)	5 (50)	
M2	17 (23)	10 (59)	7 (41)	
M4	28 (37)	12 (43)	16 (57)	
M5	14 (19)	2 (14)	12 (86)	
**Prognostic group—ELN** (%)				0.04
Favorable	12 (16)	4 (33)	8 (67)	
Intermediate	44 (59)	24 (55)	20 (45)	
Adverse	19 (25)	4 (21)	15 (79)	
**Complete remission** (%)				1.0
success	41 (55)	17 (41)	24 (59)	
failure	34 (45)	15 (44)	19 (56)	
**Relapse** (%)				0.38
yes	22 (51)	11 (50)	11 (50)	
no	19 (49)	6 (32)	13 (68)	

**Table 2:** Mutational status of *FLT3*-ITD and *NPM1* gene and miR-222 expression status in AML-NK patients stratified by the level of *GAS5* expression.

Parameter	Total (*n* = 39)	*GAS5* Expression		*p*
		*GAS5*^high^ (*n* = 16)	*GAS5*^low^ (*n* = 23)	
***FLT3*** **-ITD mutations**				0.32
present (%)	12 (31)	3 (25)	9 (75)	
absent (%)	27 (69)	13 (48)	14 (52)	
***NPM1*** **mutations**				0.21
present (%)	13 (33)	3 (25)	10 (75)	
absent (%)	26 (67)	13 (50)	13 (50)	
***FLT3*** **-ITD/*NPM1* status** **(risk group)**				0.05
*FLT3*-ITD^−^*/NPM1*^−^ (intermediate)	20 (51)	12 (60)	8 (40)	
*FLT3*-ITD^+^ (adverse)	12 (31)	3 (25)	9 (75)	
*NPM1^+^* (favorable)	7 (18)	1 (14)	6 (86)	
**miR-222 expression status**				0.40
high (%)	19 (49)	6 (32)	13 (68)	
low (%)	20 (51)	10 (50)	10 (50)	

## Data Availability

The authors confirm that the data supporting the findings of this study are available within the article, further inquiries can be directed to the corresponding author.
